# Brain death following ingestion of E‐cigarette liquid nicotine refill solution

**DOI:** 10.1002/brb3.1744

**Published:** 2020-07-28

**Authors:** Maenia Scarpino, Manuela Bonizzoli, Cecilia Lanzi, Giovanni Lanzo, Chiara Lazzeri, Giovanni Cianchi, Francesco Gambassi, Francesco Lolli, Antonello Grippo

**Affiliations:** ^1^ Neurophysiopathology Unit Neuromuscular Department AOU Careggi Florence Italy; ^2^ IRCCS Don Carlo Gnocchi Florence Italy; ^3^ Traumatic Intensive Care Unit Neuromuscolar Department AOU Careggi Florence Italy; ^4^ Clinical Toxicology Emergency Department AOU Careggi Florence Italy; ^5^ Biomedical Science Department Mario Serio University of Florence Florence Italy

**Keywords:** brain death, cotinine, e‐liquid, nicotine

## Abstract

**Background:**

The use of electronic cigarettes (e‐cigarettes) is very common worldwide. To date, an increase of nicotine intoxication following an accidental or intentional ingestion/injection of refill solution (e‐liquid) has been detected.

**Case:**

A 23‐year‐old man presented with sudden loss of consciousness, bradycardia, and respiratory muscle paralysis after intentional ingestion of e‐liquid. Early clinical data, brain computed tomography, and neurophysiological tests (electroencephalogram [EEG] and somatosensory evoked potentials [SEPs]) did not show features with a poor neurological prognostic meaning of an hypoxic encephalopathy. After 4 days, the patient showed bilateral loss of the pupillary reflex, and severe and cytotoxic edema was detected on brain magnetic resonance imaging. SEPs showed a bilateral loss of cortical responses and EEG a suppressed pattern. Nine days after the onset of coma, the patient evolved toward brain death (BD).

**Discussion:**

Because nicotine intoxication might cause respiratory muscle paralysis, without cardiac arrest (CA), it would be important to understand the mechanisms underlying brain damage and to take into account that the current neurological prognostic evidence for hypoxic–ischemic encephalopathy, based on data from patients who all experienced CA may not be reliable. Reporting cases of nicotine intoxication through e‐liquid is relevant in order to improve regulatory parameters for e‐liquid sale.

## Graphical abstract

Electronic cigarettes (e‐cigarettes), based on the vaporization of e‐liquid, a solution composed of nicotine, vegetable glycerine, and propylene glycol, have become very common worldwide and can be easily purchased online, where virtual stores are poorly monitored. This is representing a relevant health problem in the last decade, because of the increase in cases of accidental or intentional poisoning through the ingestion and/or the injection of e‐liquid composed of high‐dose nicotine. We report a case of voluntary ingestion of e‐liquid refill that caused respiratory arrest causing coma and that in the following days evolved toward brain death. This case is the first with fatal outcome in the absence of concomitant cardiac arrest. The constant reporting of cases of nicotine intoxication through e‐liquid both accidental or intentional is an important goal in order to raise the awareness of the Food and Drug Administration in improving regulatory parameters for e‐liquid and in increasing the monitoring of online stores.

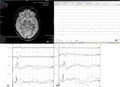

## 1 INTRODUCTION

Electronic cigarettes (e‐cigarettes), based on the vaporization of e‐liquid (a solution composed of nicotine, vegetable glycerine, and propylene glycol), have become very common worldwide. However, despite regulatory parameters, such as the maximum nicotine concentration of 20mg/ml, for e‐liquids having been proposed worldwide (Food & Drug Administration, HHS, 2016), e‐liquid solution with nicotine concentrations of up to 60mg/ml can still be easily purchased online. This has come to represent a relevant health problem in the last decade because of the increase in cases of accidental or intentional poisoning through the ingestion and/or the injection of e‐liquid. The most prevalent reported symptoms related to nicotine intoxication are tachycardia, hypertension, vomiting, diarrhea, agitation, and headache (Maessen et al., 2020). However, when high doses of nicotine are ingested and/or injected, bradycardia, hypotension, altered mental status, seizures, and, albeit rarely, death due to cardiac arrest (CA) might develop (Benowitz, 2008). To date, there is still no consensus on nicotine lethal dose. Recently, Maessen et al. (2020) reported that the minimum lethal nicotine plasma concentration was set at values between 800 and 1,600 µg/L (Maessen et al., 2020), which were higher than the previously accepted value (180 µg/L; Mayer, 2014). We report a case of severe brain injury, evolving toward brain death(BD) after nicotine intoxication, following an intentional ingestion of e‐liquid as a suicide attempt.

## 2 CASE

A 23‐year‐old man was admitted to the emergency department (ED) of Careggi Hospital, Florence, in a comatose state after the ingestion of two e‐cigarette refills. Immediately after the ingestion of the e‐liquid, the patient had a sudden loss of consciousness with vomiting, followed by bradycardia and respiratory muscle paralysis. Cardiopulmonary resuscitation was started few minutes later by a neighbor (an anesthesiologist doctor). After 20 min, the patient found unconscious but with a pulse by the emergency team of physicians, underwent orotracheal intubation and was later admitted to the ED.

**FIGURE 1:**
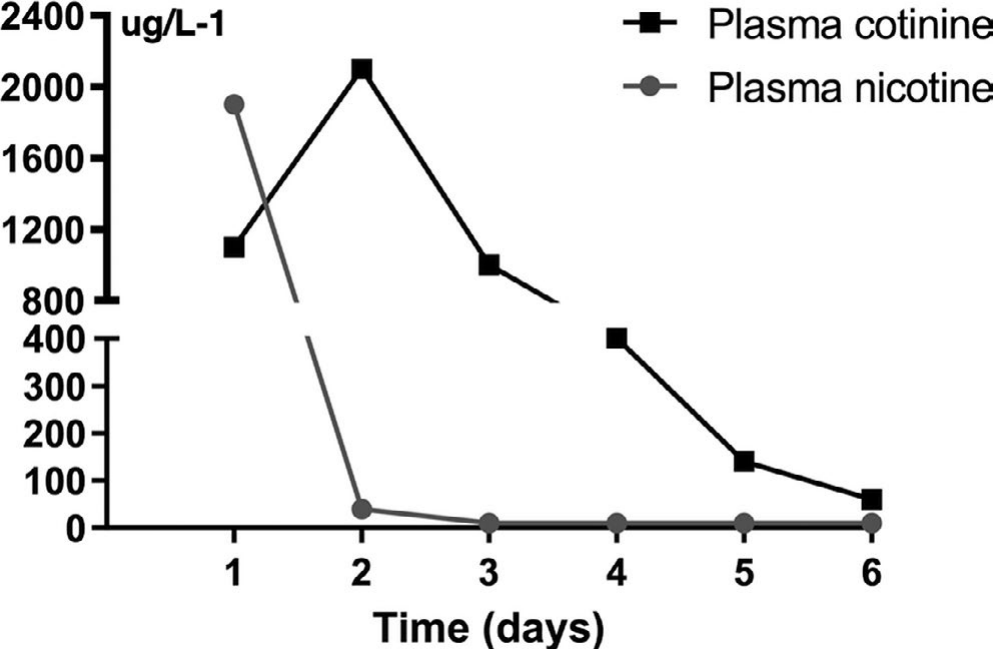
Plasmatic concentration of nicotine and cotinine measured by LC/MS‐MS technique

**FIGURE 2:**
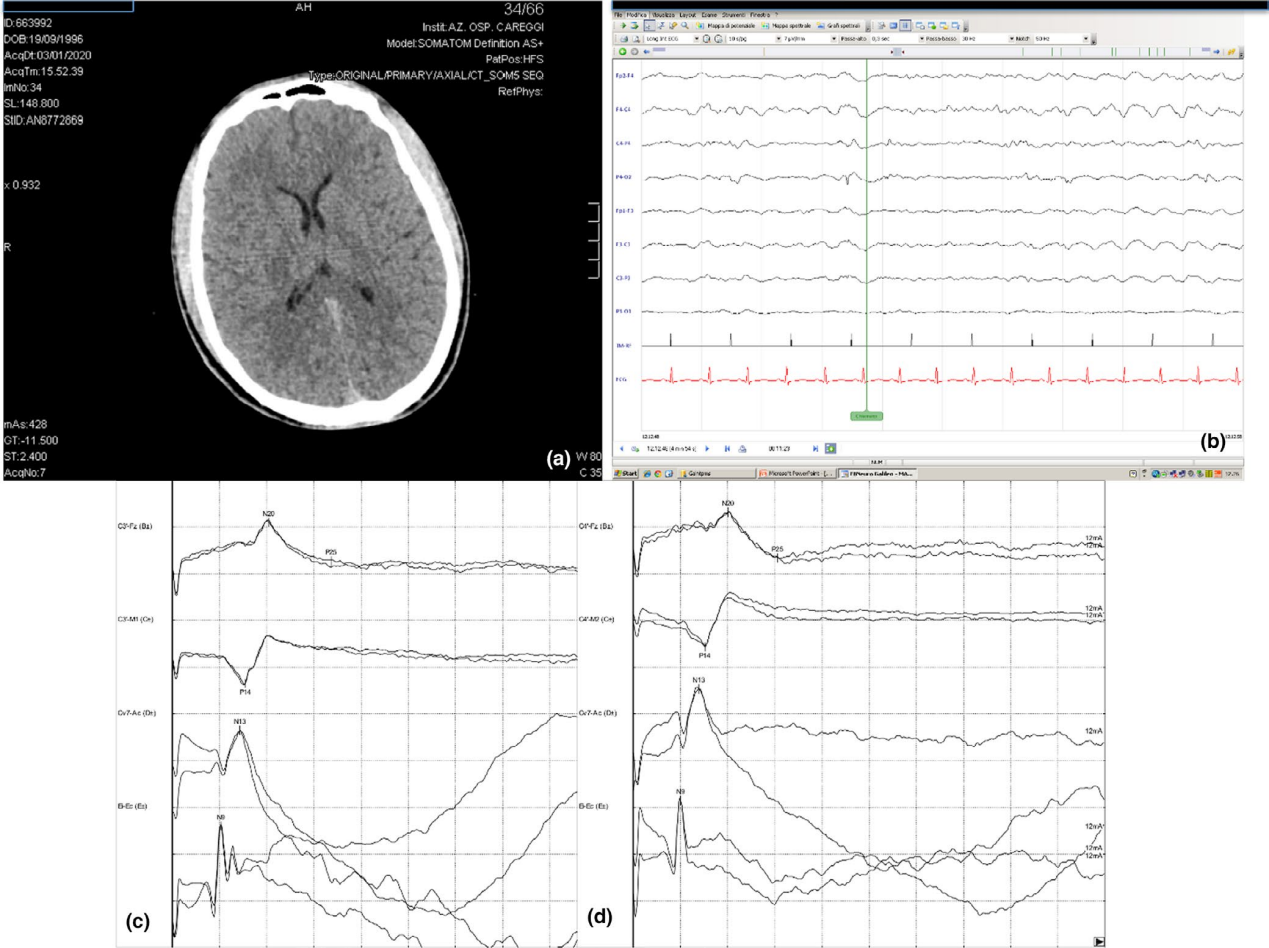
Neuroimaging and neurophysiological data within 24h after coma onset: (a) brain CT: no signs of brain edema or other signs of severe hypoxic encephalopathy: gray matter/white matter (GM/WM) ratio = 1.32; (b) EEG: continuous EEG pattern with normal voltage and with inconstant reactivity (c, d) SEPs: bilateral presence with normal voltage of cortical response (N20) (SEP pattern NN)

At the time to the arrival to the ED, the patient was hemodynamically stable with no need for vasoactive drugs (blood pressure 130/80 mm Hg, pulse 80 bpm). The blood gas analysis was as follows: pH 7.3, pCO_2_ 43 mm Hg, pO_2_ 120 mm Hg, BE: – 0.4 mmol/L, cHCO_3_: 24, SpO_2_ 97%, and FiO_2_ 0.3. He underwent gastric lavage with retrieval of a brownish viscose liquid. Liquid paraffin was administered by the same route given the oily nature of the xenobiotic ingested. The first blood sample, collected at about 2 hr postnicotine ingestion and tested for nicotine and cotinine concentration by liquid chromatography–tandem mass spectrometry (lc‐ms/ms), showed a peak value of plasmatic nicotine of 1,900 µg/L, while the cotinine peak value was 2,100 µg/L (Figure 1). No positivity to other drugs was detected. A brain computed tomography (CT) showed no signs of brain edema or other signs of severe hypoxic encephalopathy, with a gray matter/white matter (GM/WM) ratio = 1.32 (Figure 2a). The patient was thus admitted to the intensive care unit (ICU) with a Glasgow coma scale ≤ 3 but with the bilateral presence of the pupillary reflex. After the admission to the ICU, an echocardiographic assessment and electrocardiogram (ECG) were performed, showing normal findings. The oxygenation index was good (P/F > 300). During ICU stay, the patient remained hemodynamically stable and no arrhythmic events were detected on ECG monitoring. At about 7 hr after coma onset, an electroencephalogram (EEG), recorded in the absence of sedation, and somatosensory evoked potentials (SEPs) were performed, showing a nonreactive near‐continuous low‐voltage pattern and the bilateral presence with normal voltage of the cortical response (N20) (SEP pattern NN), respectively. At 24 hr after coma onset, EEG became continuous with normal voltage and with inconstant reactivity (Figure 2b), and SEPs still showed a NN pattern (Figure 2c,d).

**FIGURE 3:**
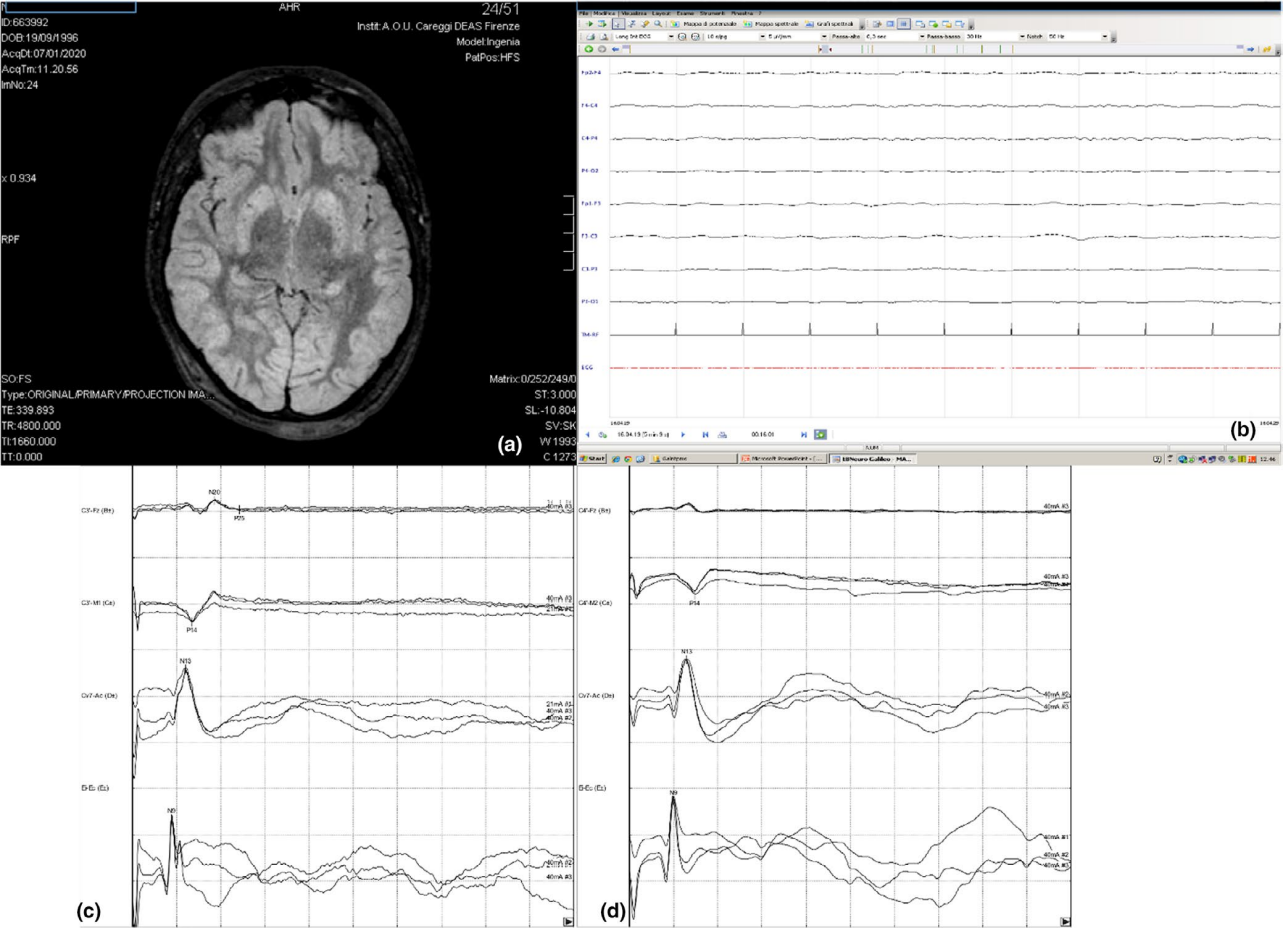
Neurophysiological and neuroimaging data four days after coma onset: (a) MRI: hyperintensity on fluid‐attenuated inversion recovery‐weighted imaging at bilateral parieto‐occipital lobes and at frontal level: (b) EEG: EEG suppressed pattern; (c, d) SEPs: bilateral deterioration of the cortical responses with a pattern pathological (c) and absent (d)

Four days after coma onset, the patient lost the pupillary reflexes. At this time, the cortical SEP responses became pathological/absent (PA; Figure 3c,d) and the EEG became suppressed and areactive (Figure 3b). On the same day, a brain magnetic resonance imaging (MRI) was performed, revealing the presence of severe and diffuse cytotoxic edema. In particular, a diffuse hyperintensity was observed bilaterally in the parieto‐occipital lobes and at the level of the frontal lobes on diffusion‐weighted imaging and on fluid‐attenuated inversion recovery‐weighted imaging (Figure 3a). A reduced diffusion measured with apparent diffusion coefficient was also detected. At last, a transcranial Doppler examination documented symmetrical flow acceleration of all arteries of the base, indicating hyperinflow. The day after, an isoelectric EEG pattern and an absent/absent (AA) cortical SEP pattern were detected in the presence, however, of the bilateral lemniscal wave, generated at the bulbar level. Nine days after coma onset, the patient showed the loss of respiratory drive and evolved toward BD.

### 2.1 Ethical statement

We confirm that we have read the journal's position on issues involved in ethical publication and affirm that this report is consistent with those guidelines.

## 3 DISCUSSION

This represents the first case of evolution toward BD after nicotine intoxication occurring in Italy and related exclusively to oral ingestion of e‐liquid. In the only previous reported Italian case (Cervellin, Luci, & Bellini, 2013), in fact the patient mixed e‐liquid with methadone and took the solution both orally and intravenously.

To date, only few cases of fatal acute intoxication with a known quantity of nicotine or precise plasma concentration have been published. Moreover, mechanisms underlying nicotine toxicity at both the central and peripheral nervous system (CNS/CNP) levels are still unclear. High doses of nicotine, as reported in our case, in whom the nicotine plasma concentration was higher than the cutoff considered to be lethal in recent literature (Maessen et al., 2020), might produce an inhibitory effect, acting through ganglionic blockade and thereby resulting in bradycardia, hypotension, and respiratory muscle paralysis (Maessen et al., 2020). At the CNS level, instead, high doses of nicotine might cause desensitization of the cholinergic receptors that normally stimulate GABAergic interneurons, resulting in reduced GABAergic activity and consequently in a reduced inhibitory effect. This mechanism could cause hyperexcitability of the CNS with an imbalance of the neurotransmitter system and a consequent increase in the release of excitatory neurotransmitters, triggering neuronal apoptosis (Dobelis, Hutton, & Lu, 2003).

In the case of oral poisoning, the prognosis usually appears to be better, since the actual bioavailability of nicotine is affected by a number of factors, such as rapid metabolism to an inactive metabolite, intense vomiting (acting as auto‐decontamination), and limited absorption in the stomach (depending on pH), even though nicotine is well absorbed in the small intestine (Maessen et al., 2020).

However, in our case, the ingested nicotine dose was high enough to cause respiratory muscle paralysis and a comatose state. Only in few other reported cases (Chen, Bright, & Trivedi, 2015; Morley, Slaughter, & Smith, 2017; Seo, Kim, & Yu, 2016), ingested nicotine dose was high enough to cause patient death, however, due to CA. In our case, there could be multiple mechanisms causing brain damage. The respiratory muscle paralysis, resulting in a reduced oxygen supply at the CNS level, could have determined a hypoxic encephalopathy. However, both the neurophysiological (EEG/SEPs) and neuroimaging data (brain CT), performed at an early stage (within 12 hr) after coma onset, did not show findings with high unfavorable neurological prognostic significance (Carrai et al., 2019; Scarpino, Carrai, et al., 2019; Scarpino, Grippo, Lanzo, & Lolli, 2018a; Scarpino, Lanzo, et al., 2018; Scarpino, Lolli, et al., 2019). In particular, the nearly continuous low‐voltage EEG pattern, observed earlier than 12 hr after coma onset, as in our case, is usually associated with a good neurological outcome (Scarpino, Carrai, et al., 2019). Moreover, patients with hypoxic–ischemic encephalopathy (HIE) evolving toward BD usually show, in the first 24 hr after coma onset, a marked alteration of SEP cortical responses (SEP pattern AA or AP; Scarpino et al., 2017), while in our case, SEP cortical responses were bilaterally normal. At last, early brain CT also did not show evidence of severe edema suggestive of an evolution toward BD (GM/WM ratio > 1.07; Scarpino et al., 2017). However, in the following few days, the patient showed a progressive worsening of all the clinical and instrumental parameters, revealing a neurological deterioration: He lost the pupillary reflexes, SEPs transitioned from a NN pattern to an AP pattern, EEG became suppressed and isoelectric in the last days, and severe brain edema was detected on MRI. This progressive neurological deterioration might be related to the development of secondary brain damage, which is sometimes observed in patients with HIE after CA, even though, in our case, early clinical and instrumental data did not show features suggesting the occurrence of secondary brain damage (Scarpino, Carrai, et al., 2019). However, it is possible that nicotine intoxication, having caused only respiratory muscle paralysis, without CA, might have triggered mechanisms of hypoxic brain damage that could be different from those of classic HIE, and, for this reason, the current neurological prognostic evidence for HIE may not be reliable, since they were based on data from patients who all experienced CA. At last, besides the hypoxic damage, we cannot exclude that high doses of nicotine could have also caused direct neuronal apoptosis, secondary to an increase in the release of excitatory neurotransmitters at the CNS level (Dobelis et al., 2003). At the same time, nicotine, binding to specific nicotinic receptors and consequently causing an increase in acetylcholine release and depolarization of cerebral microvascular muscles, might cause diffuse vasoconstriction and thrombosis (Maessen et al., 2020). These phenomena, causing severe and diffuse brain ischemia, might have determined a worsening of the brain edema. However, in our opinion, brain damage due to mechanisms related to the direct action of nicotine on the CNS would be less likely, because nicotine usually shows early action with a rapid transition to its inactive metabolite (Figure 1); consequently, its neurological effects should also be evident at an early stage after ingestion/injection (Maessen et al., 2020), which is different from our case. At last, a large literature suggests a neuroprotective effect of nicotine, mainly in chronic neurological degenerative condition such as cognitive deterioration (Alkadhi, 2018), Parkinson's disease (Quik, Bordia, Zhang, & Perez, 2015), and Tourette's syndrome (Quik, Bordia, Zhang, & Perez, 2014). However, this protective effect, as well as central or peripheral nervous system stimulation with arousal and increase in heart rate or blood pressure, is induced by low dose of nicotine, different from our case.

## 4 CONCLUSION

The constant reporting of cases of nicotine intoxication through e‐liquid both accidental or intentional is an important goal in order to raise the awareness of the Food and Drug Administration in improving regulatory parameters for e‐liquid and in increasing the monitoring of online stores.

Because nicotine intoxication might cause respiratory muscle paralysis, without CA, it would be important to understand the mechanisms underlying brain damage and to take into account that the current neurological prognostic evidence for HIE may not be reliable.

## CONFLICT OF INTEREST

None of the authors has any conflict of interest to disclose.

## AUTHOR CONTRIBUTIONS

Maenia Scarpino, Manuela Bonizzoli, Cecilia Lanzi, Chiara Lazzeri, Giovanni Cianchi, and Francesco Gambassi participated in the clinical practice, including diagnosis, treatment, consultation, and during the ICU stay of the patient. Giovanni Lanzo, Cecilia Lanzi, and Chiara Lazzeri contributed to the acquisition of data. Maenia Scarpino, Cecilia Lanzi, and Chiara Lazzeri contributed to the analysis of data. Maenia Scarpino wrote the manuscript. Antonello Grippo and Francesco Lolli revised the manuscript. All authors approved the final version of the manuscript.

### Peer Review

The peer review history for this article is available at https://publons.com/publon/10.1002/brb3.1744.

## Data Availability

The data that support the findings of this study are available from the corresponding author upon reasonable request.
